# Fabrication of black-gold coatings by glancing angle deposition with sputtering

**DOI:** 10.3762/bjnano.8.46

**Published:** 2017-02-14

**Authors:** Alan Vitrey, Rafael Alvarez, Alberto Palmero, María Ujué González, José Miguel García-Martín

**Affiliations:** 1 IMM-Instituto de Microelectronica de Madrid (CNM-CSIC), Isaac Newton 8, Tres Cantos 28760, Madrid, Spain; 2 Instituto de Ciencia de Materiales de Sevilla (CSIC-US), Americo Vespucio 49, Seville 41092, Spain

**Keywords:** black coatings, black metals, glancing angle deposition, light absorption, sputtering

## Abstract

The fabrication of black-gold coatings using sputtering is reported here. Glancing angle deposition with a rotating substrate is needed to obtain vertical nanostructures. Enhanced light absorption is obtained in the samples prepared in the ballistic regime with high tilt angles. Under these conditions the diameter distribution of the nanostructures is centered at about 60 nm and the standard deviation is large enough to obtain black-metal behavior in the visible range.

## Introduction

Black-metal coatings are metallic coatings that exhibit a high absorption in a certain region of the electromagnetic spectra. They are of interest in a wide range of applications [1–7] such as radiative heat exchangers, solar energy absorbers, electrodes of photovoltaic cells, separators to avoid cross effects in optical devices, thermal light emitters and electrodes of sensor or biosensors. In particular, gold is frequently used due to its high resistance to oxidation. As the spectrum of the solar radiation exhibits maximum irradiance in the visible range, finding a suitable method to produce black-metal coatings in the visible range is of the utmost importance for some of the abovementioned applications that require conducting behavior.

Physical vapor deposition (PVD) techniques are used to manufacture high-purity thin-film coatings in an environmentally friendly manner (no chemicals are involved, therefore no recycling problems are associated) onto any kind of substrates (e.g., conductor/isolator, flexible/rigid). By employing glancing angle deposition (GLAD), nanostructured coatings can be produced onto flat substrates, taking advantage of atomic shadowing effects. Although the nanostructures fabricated with evaporation methods (such as thermal evaporation or electron-beam evaporation) exhibit very well defined shapes and impressive homogeneity due to their almost punctual material sources, they cannot be scaled-up to mass production. Consequently, from an industrial point of view, sputtering is the best choice.

From the pioneering work of GLAD sputtering [8], the importance of “low-pressure, long-throw” deposition was stated, i.e., a collimated flux of sputtered atoms was needed to obtain nanostructure formation. In the last few years, we have followed a systematic approach to study the growth regimes [9–11] and the deposition rate [12] of the nanostructured coatings prepared by GLAD sputtering, pinpointing the role of the ballistic atoms (i.e., those that preserve their directionality when they travel from the source to the substrate) to achieve such effect. In this new work, we report the fabrication of black-gold coatings in the visible range via GLAD sputtering by rotating the substrate during the deposition. It will be shown that we have achieved over 85% of absorption between 400 nm and 700 nm when silicon substrates are used.

## Experimental

**Figure 1:**
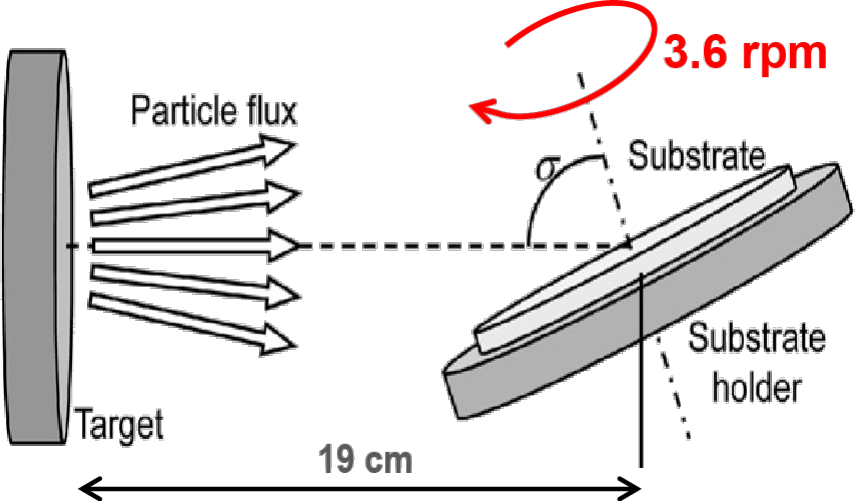
Scheme of the sputtering process with glancing angle deposition and rotating substrate.

The fabrication of the nanostructured coatings has been carried out at room temperature in an UHV chamber (base pressure in the range of 10^−9^ mbar) using a magnetron source from AJA with a gold target (3.8 cm diameter). Argon was the sputter gas and the pressure during the deposition was 1.5 × 10^−3^ mbar, which was the lowest value that allowed for maintaining a stable plasma and for warranting that the deposition took place in the ballistic regime. The target-to-substrate distance was 19 cm, the substrate rotated at 3.6 rpm and the power and the deposition time were kept constant at 100 W and 1800 s, respectively. Two different monocrystalline substrates have been used: opaque Si(001) and transparent MgO(001). The tilt angle, σ, was varied from 75 to 87°. A scheme of the set-up is depicted in Figure 1. For the sake of comparison, continuous thin films were also fabricated using the standard configuration with the substrate being parallel to the target, i.e., σ = 0°, the same power (100 W) and the equivalent deposition time to get the same amount of material per area as in the GLAD configuration.

The morphological characterization has been obtained by field emission scanning electron microscopy (FESEM), on a Hitachi equipment working at 5 kV, and atomic force microscopy (AFM), on a Dimension Icon microscope from Bruker with super-sharp tips (radius about 3 nm) to minimize the inherent convolution with the shape of the probe.

In order to study the optical behavior, spectral reflectance and transmittance measurements have been performed using unpolarized light with almost normal incidence in the VIS–nIR range and an Andor Shamrock spectrometer.

## Results and Discussion

**Figure 2:**
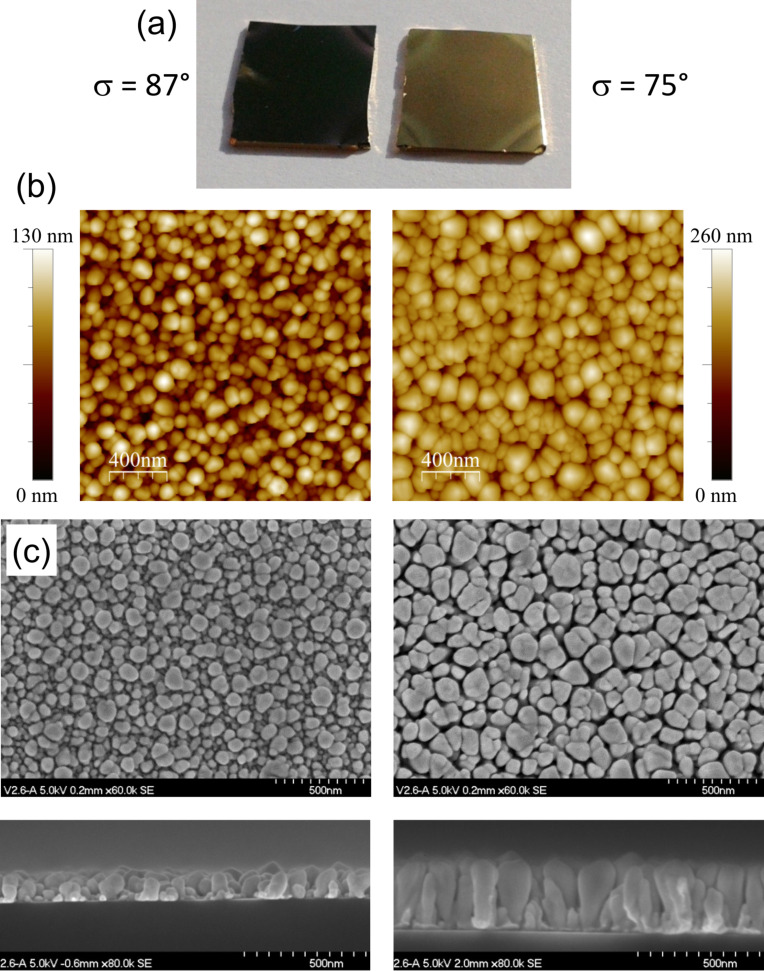
(a) Photograph, (b) AFM images, and (c) SEM images (upper row: top view, lower row: fracture cross section) of two Au samples prepared onto Si substrates with σ = 87° (left column) and σ = 75° (right column).

Figure 2a shows a photograph of two samples prepared onto Si(001) substrates with 1 cm^2^ area: the one on the left was prepared with σ = 87° and exhibits black color, whereas that on the right was prepared with 75° tilt and shows golden color. These samples present different morphologies, as it is shown in Figure 2b and Figure 2c with AFM and FESEM images, respectively. As a result of the shadowing mechanism at the nanoscale induced by the ballistic regime (due to the low pressure used) and the high-tilt configuration (σ > 70°), instead of continuous thin films, nanostructures are formed. Due to the substrate rotation during the glancing angle deposition, these nanostructures display axial symmetry, with the axis in the vertical direction. It can be seen that, on average, the black 87° sample has shorter nanostructures than the golden 75° one: the highest nanostructures on the former sample are about 130 nm height, whilst those on the latter reach 260 nm. Moreover, with a careful inspection it can also be seen that the height distribution of the nanostructures obtained at σ = 87° is wider than the height distribution of those obtained at σ = 75°.

**Figure 3:**
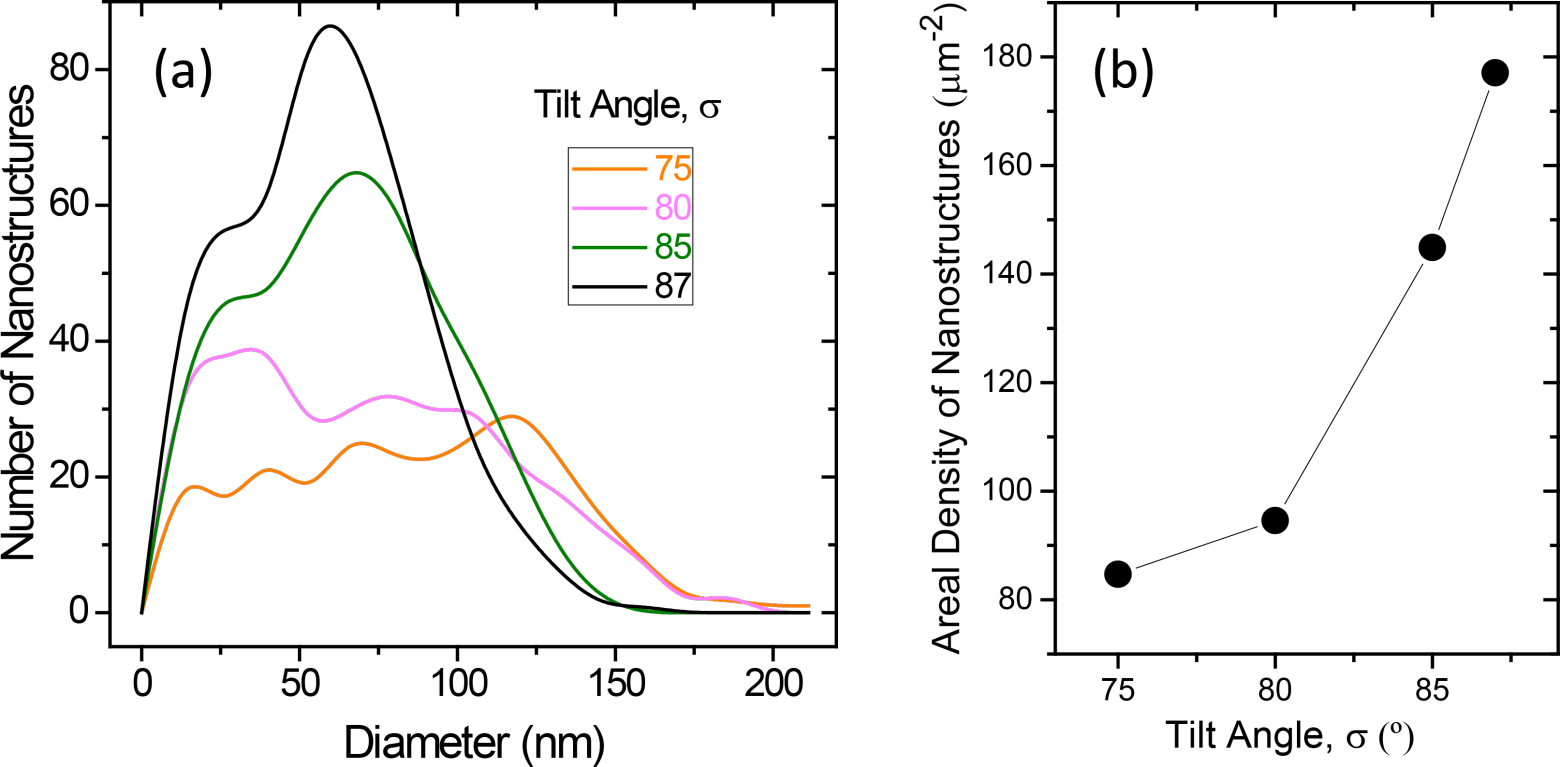
(a) Diameter distribution obtained from top-view SEM images for the Au samples prepared onto Si substrates with different tilt angles. (b) Areal density of nanostructures of the same samples.

Furthermore, from the top-view SEM images the diameter size distribution can be obtained, and it is plotted in Figure 3a. The samples prepared with σ = 75° and σ = 80°, both exhibiting golden color, have wide distributions with a plateau shape and a significant number of nanostructures with diameters above 130 nm. On clear contrast, the samples prepared with σ = 85° and 87°, both black, have narrower distributions, with a well-defined peak corresponding to 70 nm and 60 nm diameter, respectively. Figure 3b shows the dependence of the areal density of nanostructures on the tilt angle, also deduced from the SEM images. When the tilt angle increases, the number of nanostructures per area also increases. Summarizing the information obtained from Figure 2 and Figure 3, high tilt angles produce more nanostructures, with narrower lateral size distribution and with a strongly non-uniform height distribution. On the contrary, low tilt angles generate less nanostructures, with wider lateral size distribution and more uniform in height.

**Figure 4:**
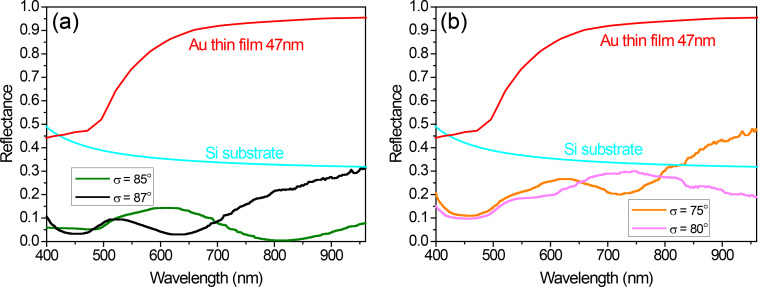
Reflectance spectra of nanostructured Au samples exhibiting black color (a) and golden color (b). For the sake of comparison, the calculated spectra of a bare Si substrate (cyan) and a continuous Au thin film (red) have been also included.

These important differences in the morphology of the samples are responsible for their different appearance shown in Figure 2 and their distinctive spectral behavior presented in Figure 4, where the reflectance of the samples with black and golden color is plotted. For the sake of comparison, the calculated reflectance of a bare Si substrate and that of a sample with a continuous Au layer with 47 nm thickness (obtained with the deposition time that provides the equivalent material per area as in the case of GLAD deposition) are also included (the dielectric constants for Si and Au in the calculations were taken from [13] and [14], respectively). The most significant fact in Figure 4a is that the nanostructured samples deposited at high tilt angle exhibit low reflectance in the visible range (400–700 nm), less than 10% for the sample prepared with σ = 87° and less than 15% for that prepared with σ = 85°. Taking into account that the optical properties of gold nanostructures are dominated by the existence of localized surface plasmon resonances (LSPRs), and that these LSPRs induce enhanced scattering, it is reasonable to assume that such low reflectance is due to the combined effect of the LSPRs and the light trapping associated with multiple scattering that arises when those nanostructures are closely packed forming a non-periodic, non-uniformly sized array [15–16]. In particular, the high number of nanostructures and their broad height distribution favors multiple scattering processes, improving light trapping and increasing absorption, therefore resulting in a uniform low reflectivity and the black color appearance. On the other hand, the reduced number of nanostructures exhibited by the samples prepared with σ = 75° and 80°, together with their broader lateral size distribution and their height uniformity, do not provide such an efficient light trapping (Figure 4b). It can be seen that the reflectance values shown by these samples are not as low as those obtained in the samples prepared with 87° and 85° tilt angle. Finally, the only common feature for all the nanostructured samples and the continuous film is a decrease in the reflectance at wavelengths below 500 nm, which is due to an interband transition in gold [17] and it is responsible of the golden color of continuous and bulk gold. The values of this decrease are, however, quite different. The values are very small for the high tilt angle deposition samples, which therefore present a uniform low reflectivity in the visible range and look black. But they are more noticeable in the samples with σ = 75° and 80°, which then preserve the golden color, although dull because of the reduced reflectivity.

**Figure 5:**
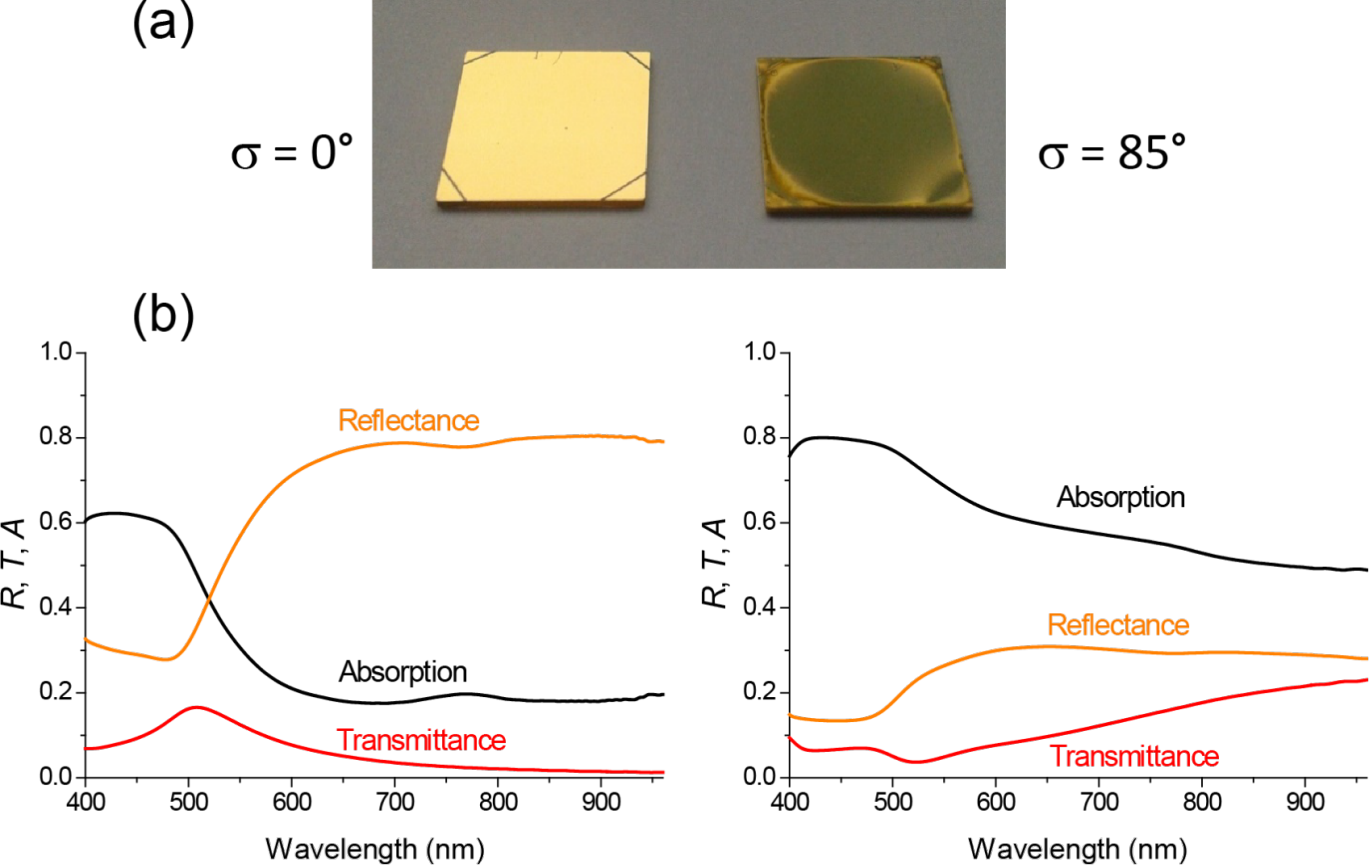
(a) Photograph of two samples prepared onto MgO substrates: a continuous Au thin film prepared with σ = 0° (left) and a nanostructured Au sample prepared with σ = 85° (right). (b) Transmittance, reflectance and absorption spectra of those samples.

The black-metal behavior has been also obtained with transparent substrates. Having in mind a future scalability of the process, from an industrial point of view it is better to use the smaller tilt angle that is able to give rise to black-metal samples. Hence, 85° has been our choice for this proof of concept. Figure 5a shows a photograph of two gold samples prepared onto MgO(001) substrates with 1 cm^2^ area: The one on the left was prepared without tilt angle and as a consequence is a continuous thin film, whereas that on the right was prepared with σ = 85° and therefore is nanostructured. The continuous sample exhibits the characteristic golden color of the noble metal, whereas the nanostructured sample shows a much darker color. Their optical spectra are shown in Figure 5b. The transmittance (*T*) and the reflectance (*R*) have been measured directly, whereas the absorption (*A*) has been deduced taken into account that *T* + *R* + *A* = 1 (strictly speaking, in the nanostructured sample the magnitude A that we are deducing is extinction = absorption + scattering instead of merely absorption). As it was explained previously, both samples exhibit a decrease of the reflectance for wavelengths below 500 nm due to a characteristic interband transition in gold. Above 500 nm, the evolution is quite different. The continuous film shows the characteristic reflecting behavior of metallic films, related to the contribution of the free electrons (Drude) to the dielectric constant. The reflectance of this sample goes up to 80%. In the nanostructured sample, on the other hand, the same contribution of the free electrons to the dielectric constant is responsible for the appearance of LSPRs, which as discussed above induce enhanced multiple scattering and light-trapping effects for the particular size distribution and separation among nanostructures occurring in the nanostructured films obtained at high tilt angle. As a result, the nanostructured sample exhibits absorption above 60% over the whole visible-light range, slightly decreasing to 50% in the nIR region.

## Conclusion

It has been shown that black-gold coatings in the visible range can be fabricated by means of sputtering using glancing angle deposition with substrate rotation. The coatings are made of columnar nanostructures that are produced due to shadowing effects in the ballistic regime of sputtering. In order to obtain enhanced light absorption in the visible range, high tilt angle is needed, which gives rise to a diameter distribution of the nanostructures centered at about 60 nm.
